# Static Anthropometric Characteristics of Bangladeshi Vehicle Driver: A Case Study

**DOI:** 10.1155/2016/1329612

**Published:** 2016-08-07

**Authors:** P. K. Halder, E. Sarker

**Affiliations:** ^1^Department of Industrial and Production Engineering, Jessore University of Science and Technology, Jessore 7408, Bangladesh; ^2^Hajee Mohammad Danesh Science & Technology University, Dinajpur 5200, Bangladesh

## Abstract

*Background*. Inappropriate design of sitting furniture and working equipment causes the serious musculoskeletal injuries and various pains as well as reducing working efficiency. Uncomfortable sitting posture in prolonged driving in Bangladesh is an issue to be solved immediately. Therefore, anthropometric databank of user population is significantly essential for the suitable dimensional design for avoiding these remarkable problems.* Methods*. This study analyses the anthropometric data of the Bangladeshi male vehicle driver aged between 30 and 60 years. A total of 210 Bangladeshi healthy drivers are considered for 15 anthropometric measurements and compared with the similar anthropometrics of other nationalities.* Results.* The mean stature and sitting height erect of Bangladeshi driver are 1645 mm and 843 mm, respectively. The mean of body mass index (BMI) of the drivers is 26.09 kg/m^2^, which indicates that the drivers are overweight. The mean stature of Bangladeshi driver is 17 mm shorter than the driver of Korea and 115 mm shorter than the driver of Iran.* Conclusion*. There are substantial differences between the body dimensions of Bangladeshi driver and similar dimensions of other countries. In comparison, Bangladeshi driver is found to be the shortest compared with the sample of other nationalities.

## 1. Introduction

The mismatch between the seat measurements and the body dimensions of the driver causes various physical problems like discomfort, pain, and disorders in neck, shoulder, back, arm, hand, and wrist which can lead to musculoskeletal diseases [1]. This variation and uncomfortable sitting posture also affect the performance of the driver due to prolonged driving; hence, they loss their driving interest. Some of the early researches have described the improper seat design as the root cause of neck, upper back, and low back pain for the school children [2–5]. Chakrabortty et al. showed that the physical problems of patients due to improper hospital bed are related to their anthropometric factors [6]. In addition, Mououdi and Choobineh [7] and Jeong and Park [8] have provided the anthropometric data of school children and found the discrepancy with furniture measurements. Therefore, useful ergonomic measures need to be considered for avoiding these problems. Currently, Shah et al. [9], Odunaiya et al. [10], and Hoque et al. [11] have been given some attention to design ergonomically correct furniture for university students. Several studies present the anthropometric data of different countries of population of different classes including the 24 static anthropometric measurements of Portuguese adult worker [12], the measurements of 36 body dimensions of Singaporean and Indonesian populations [13], the anthropometric characteristics of northeastern Indian male agricultural workers [14] and female farm workers [15], the anthropometric data of Taiwanese women [16], Turkish populations [17], the anthropometric data of Iranian guidance school students aged 12–14 years [18] and Iranian primary school children of age between 6 and 11 years [19], the anthropometric study of the Malaysian population [20], and the Thai population [21] anthropometric measurement of Schober's test in normal Taiwanese population [22].

Some researchers, Chimote and Gupta [23], Tan et al. [24], and Mohamad et al. [25], have investigated the mismatch between driver's seat measurements and body dimensions that lead to various physical problems. Mohamad et al. [26] have proposed car seat dimensions for Malaysian male and female drivers based on their anthropometry for comfortable driving. But it is so pathetic that in Bangladesh not much research work is carried out for driver's seat. Only, Mahamud et al. [27] have investigated the mismatch between the 4 anthropometric dimensions of 30 Bangladeshi drivers, aged between 30 and 50 years and corresponding seat measurements of Tata. So, there exists a huge research scope in designing ergonomically correct vehicle driver's seat in Bangladesh which would improve sitting posture to enhance drivers comfort and to reduce physical injuries. This study focuses on the development of anthropometric data of the Bangladeshi vehicle drivers for the designer to apply the anthropometry for avoiding fatigue and ensuring comfort level of drivers.

## 2. Samples and Methodology

**Figure 1:**
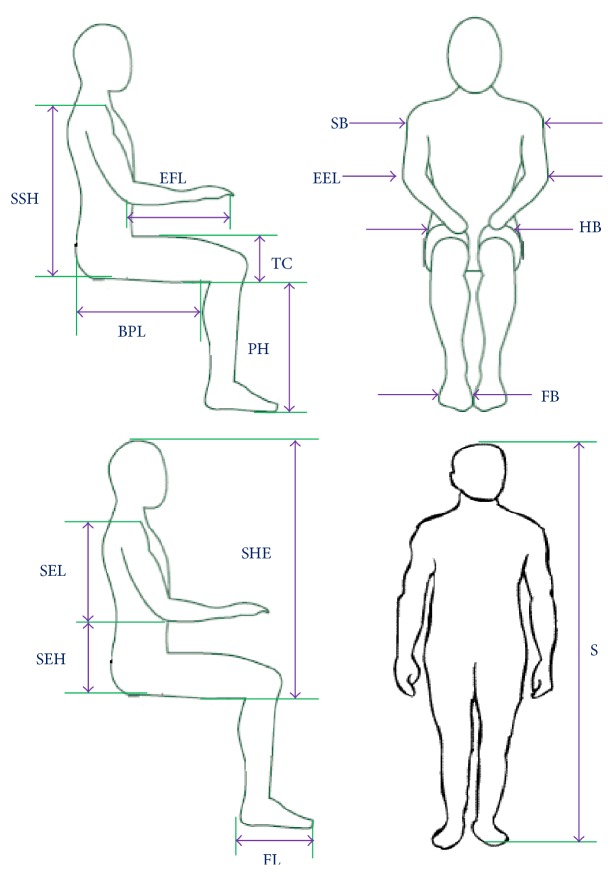
Anthropometric measurements of driver. PH: popliteal height, SHE: sitting height erect, SEH: sitting elbow height, TC: thigh clearance, BPL: buttock popliteal length, EEL: elbow to elbow length, HB: hip breadth, SSH: sitting shoulder height, SB: shoulder breadth, SEL: shoulder elbow length, EFL: elbow fingertip length, FB: foot breadth, FL: foot length, and S: stature.

This study includes a measurement of 15 sets of anthropometric data and calculation of 3 anthropometric indices of 210 Bangladeshi vehicle drivers including 130 truck drivers and 80 bus drivers with mean age of 41.00 ± 5.99 years. The participated driver sample was selected arbitrarily from central storage depot in Khulna, Bangladesh. The anthropometric data for this research were measured according to Abeysekera [28] and Pheasant and Haslegrave [29] as displayed in Figure 1. A traditional anthropometer, a measuring tape, and a standard weight measuring scale were used to measure the anthropometric dimensions and weight, respectively.

In addition, a statistical software named OriginPro 2016 was used to calculate mean value, standard deviation (St. Dev.), standard error of mean (SEM), coefficient of variation (CV), and percentile value of the body measurements. Among the indices, body surface area (BSA) was calculated by Du Bois formula [30] as shown as follows:

(1) $$\begin{array}{c} BSA=\left(\;{body\;\;weight}^{0.425}\times {stature}^{0.725}\;\right)\times 0.007184. \end{array}$$

On the other hand, relative sitting height (RSH) and body mass index (BMI) were calculated according to (2) and (3), respectively:

(2) RSH=sitting  heightstature,

(3) BMI=body  weightstature×stature.

## 3. Result and Discussion

### 3.1. Anthropometric Data Development and Analysis

The descriptive and inferential statistics of the anthropometric characteristics and indices are depicted in Table 1. The means of stature, sitting height erect, and body mass of the sample are 1645 ± 70.4 mm, 843 ± 34.6 mm, and 70.63 ± 9.04 kg respectively. In addition, the mean of sitting popliteal height is 433.7 ± 25 mm and that of the buttock popliteal length is 438.3 ± 30.1 mm. Moreover, the mean of shoulder elbow length is 358 ± 26.3 mm, while the mean of sitting elbow height is 235 ± 21.3 mm.

**Figure 2:**
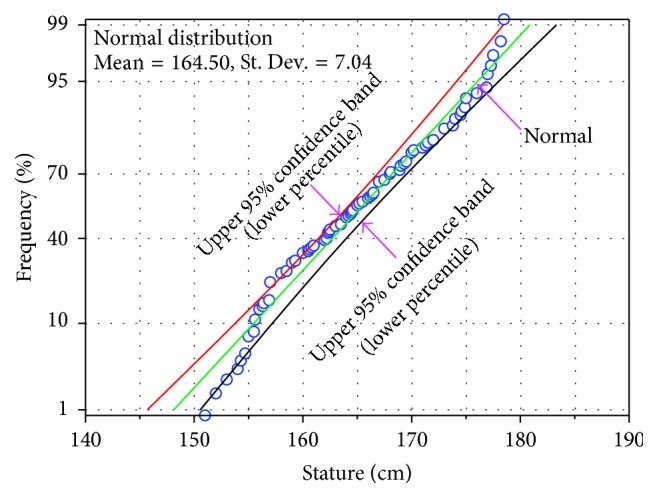
Distribution of stature of the sample.

Standard deviation of the anthropometric dimensions varies from 7.9 to 70.4 mm. The highest St. Dev. of stature indicates that the maximum number of sample deviates from their mean of stature measurement. On the other hand, lowest St. Dev. of foot breadth means most of the samples are close to its mean. The 5th, 50th, and 95th percentile values of stature are 1549.9 mm, 1641 mm, and 1770 mm, respectively, which indicates that 5% of the population have a height below 1549.9 mm and 5% of the population have a height above 1770 mm. On the other hand, 50% of the populations have a height almost the same to the mean of the stature. The normal distribution of stature measurement of the sample is presented in Figure 2. At 5% level, all the data was taken significantly from the normally distributed population. It is estimated that almost 60% of the sample is in the mean range of 1645 ± 70.4 mm. On the contrary, approximately 18.33% of the sample has the value over higher limit (1715.4 mm) and 21.67% of the sample has the value below lower limit (1544.6 mm).

The value of SEM for the anthropometric measurements and indices varies from 0.001 to 0.82 indicating the precision how the mean of the sample closes to the mean of the total population. In this study, weight has the highest SEM and RSH has the lowest SEM. The CV measures the relative variability to the mean and varies from 2.88 to 12.79% in this study.

Skewness indicates the measurement of asymmetry of a distribution around its mean value. Foot breadth shows the highest positive value (0.38) indicating the distribution of tail extended more towards its positive value from its mean value (Table 1). On the other hand, sitting shoulder height with the highest negative value (−0.57) presents the distribution of tail extended more towards its negative value from its mean value. Kurtosis measures the flatness of a distribution. Kurtosis with a value greater than zero indicates the too much peak distribution, whereas Kurtosis with negative value means that the distribution is too much flat.

The ratios* E*1 and* E*2 are calculated for all the anthropometric measurements which indicate the necessity of modifying the design of sitting equipment based on stature.* E*1 is the ratio of the mean of each anthropometric measurement to the mean of the stature, while* E*2 is the ratio of the SD of each anthropometric measurement to the SD of the stature. Table 1 indicates that the values of* E*1 vary within the ranges of 0.06 to 0.51, whereas the values of* E*2 are calculated from 0.11 to 0.51. Sitting height shows the highest value of* E*1 and foot breadth shows the lowest value.

**Figure 3:**
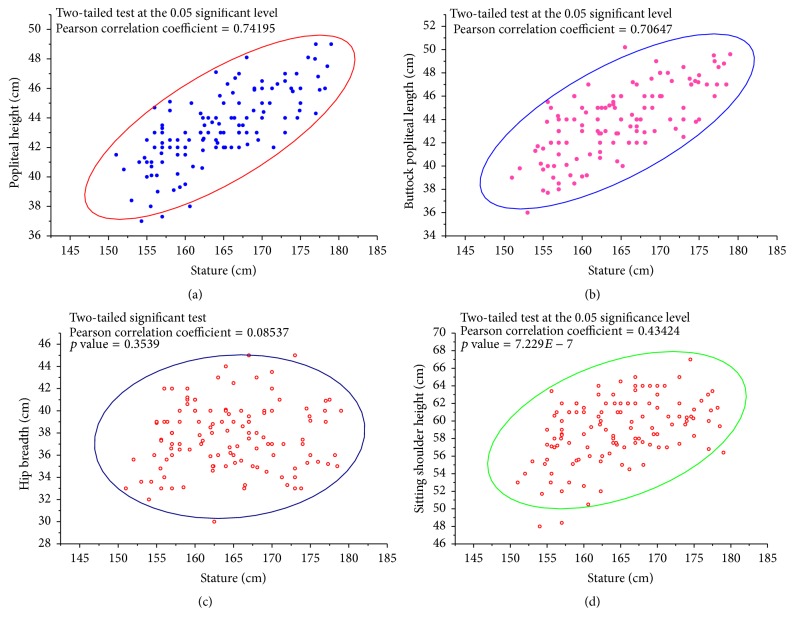
Frequency distribution of the most important anthropometric dimensions related to seat design.

Frisancho [39] suggests that BMI within the ranges of 18.50–25.00 kg/m^2^ is considered as normal. In this study, the mean of BMI is 26.09 ± 2.86 kg/m^2^ indicating that the drivers are slightly overweight. In addition, it is noticeable that almost 5% of the sample is obese having BMI value over 30 kg/m^2^ and over 45% of the sample is overweight. On the contrary, the percentage of underweight population is approximately below 5% of the sample. Hence, slightly over 40% of the sample is neither overweight nor underweight. According to Mosteller [40] average normal body surface area ranges from 1.07 to 1.92 m^2^ depending on the gender and age of the population. Table 1 reveals that mean of BSA is 1.77 ± 0.13 m^2^ indicating the normal condition of the sample. As an exception to this, the percentile values depict that approximately 95% of the sample is in the range of normal situation and 5% of the sample exceeds the normal range. The mean of relative sitting height of the sample is 0.51 ± 0.01 and the 5th, 50th, and 95th percentile values are 0.49, 0.51, and 0.53, respectively. According to Pheasant and Haslegrave [29] all the percentile values indicate that the sample is long-legged. Mahamud et al. [27] indicated that popliteal height, popliteal length, hip breadth, and sitting shoulder height are the most significant anthropometries for determining the most suitable seat measurements. A scatter plot of these four body dimensions against the stature of the sample is illustrated in Figure 3 enclosing 95% of the sample by an ellipse. The correlation is significant at the level of 5%.

However, small sample size is the main constraint of developing a reliable anthropometric databank in any region. The measurements of large sample size approximate the actual data of the total population. Therefore, as much as large sample size is required for developing a comprehensive anthropometric database for future studies and design problem.

### 3.2. Comparative Study with Other Country

In this study, anthropometric data of Bangladeshi vehicle driver is compared to the similar data of the different vehicle driver of several countries such as Nigeria, Korea, Iran, and China. The comparative study of stature measurement presented in Table 2 shows that Bangladeshi vehicle driver has the shortest stature measurement (approximately <17 to 115 mm) compared to the other countries. The highest mean of stature (1760 mm) is recorded for Iranian Shoka vehicle driver.

Table 3 illustrates that the Chinese driver has the highest sitting height, while the Korean car driver belong the highest shoulder height in sitting position that indicates high backrest height requirement for proper sitting posture. Besides, Bangladeshi driver shows the thickest thigh and longest buttock popliteal height in sitting position indicating high seat height for comfort.

**Figure 4:**
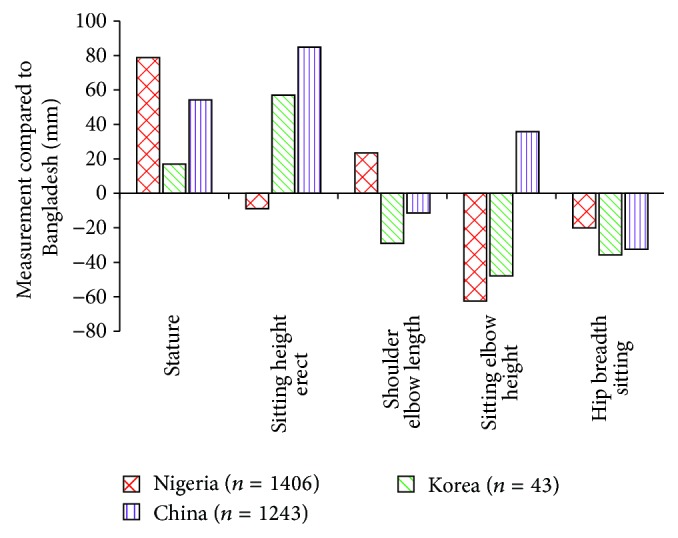
Differences of mean of selected body dimensions from different countries compared to Bangladesh.

Table 3 also indicates that the Nigerian taxicab driver has the highest shoulder elbow length, whereas the Chinese driver has the highest elbow height in sitting position. In addition, highest hip breadth measurement is found for Bangladeshi vehicle driver which represents the requirement of wide seat for ergonomical fit. The high value of BMI of Bangladeshi drivers signifies that they are fatter than other nationalities. Compared to Bangladeshi driver, the Nigerian driver is about 78.8 mm longer and the China driver is almost 54.2 mm longer as depicted in Figure 4. Besides, the sitting elbow height for the Nigerian driver is approximately 62.5 mm shorter and that for the China driver is nearly 35.8 mm longer than the Bangladeshi driver.

**Figure 5:**
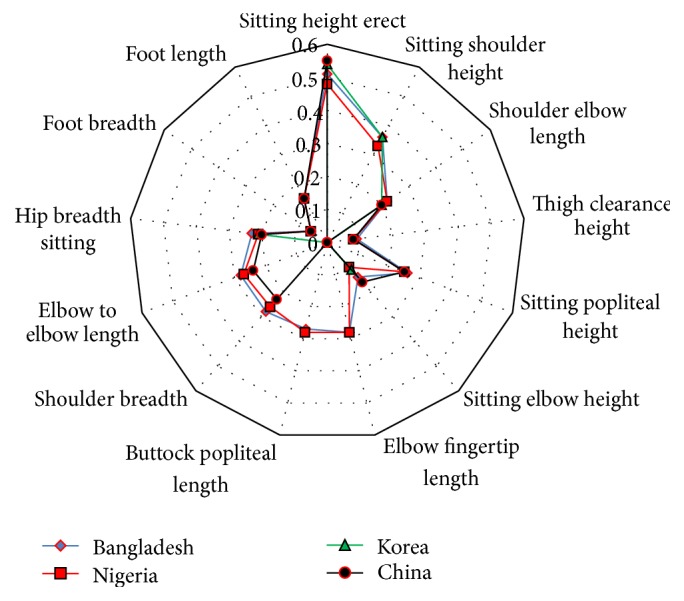
Variation in* E*1 value of anthropometric dimensions for different countries.

The values of* E*1 for available anthropometric measurements found in literature related to driver's anthropometry are calculated and the variation among the countries is presented in Figure 5. It is clearly depicted that the values of* E*1 for the most of the anthropometric measurement are approximately the same for the nationalities. The highest values of* E*1 for sitting height erect and sitting elbow height are found for the Chinese driver and the lowest values are for the Nigerian driver. Bangladeshi driver has the highest value of* E*1 for elbow to elbow length and shoulder breadth, while the Chinese driver has the lowest value. Moreover, the highest value of* E*1 for sitting shoulder height is recorded for both Bangladesh and Korea.

The anthropometric characteristics considered in this study can be comparable to the body dimensions from the list described in ISO 7250 [41]. According to the ISO standard, the design midrange of stature for the Asian region is 1698 mm, while the mean of stature in this study is 1645 mm. Although most of the body dimensions found in this study are slightly lower than the ISO midrange standard of Asian region, shoulder elbow length, sitting popliteal height, shoulder breadth, and foot breadth have marginally higher value compared to the standard midrange.

## 4. Conclusion

This study develops the first anthropometric database of Bangladeshi vehicle driver. Fifteen anthropometric characteristics and 3 anthropometric indices of 210 vehicle drivers were measured and various descriptive statistics were calculated. These measurements were also compared with those of the other countries available in the literature, though the sample size and the geographic conditions of the studies were different. However, a significant difference among the characteristics was found. In summary, the Bangladeshi drivers were the shortest compared to the other studies and had the widest shoulders and hips. This study was the preliminary step for developing anthropometric database with small sample size. However, a large sample size could develop accurate and precise anthropometric databank of Bangladeshi vehicle driver for commercial design application for future research.

## Figures and Tables

**Table 1:** Anthropometric dimensions of Bangladeshi vehicle drivers.

Measurements	Mean	St. Dev.	Percentile			SEM	CV (%)	Skewness	Kurtosis	*E*1	*E*2
			5th	50th	95th						
Stature	1645	70.4	1549.9	1641	1770	0.64	4.28	0.24	−0.90	—	—
Sitting height erect	843	34.6	784.9	845	900	0.32	4.11	−0.26	−0.55	0.51	0.49
Sitting shoulder height	589.6	35.8	525.7	592.5	640	0.33	6.08	−0.57	0.35	0.36	0.51
Shoulder elbow length	358	26.3	312.9	359	405.1	0.24	7.33	0.16	0.11	0.22	0.37
Thigh clearance height	146.3	18.1	115	150	175.1	0.17	12.37	−0.11	−0.59	0.09	0.26
Sitting popliteal height	433.7	25	391	430	471.2	0.23	5.76	−0.10	−0.13	0.26	0.36
Sitting elbow height	235	21.3	200	235	265.1	0.19	9.07	−0.49	0.42	0.14	0.30
Elbow fingertip length	461.5	28.4	412.9	461.5	505.1	0.26	6.15	−0.28	−0.57	0.28	0.40
Buttock popliteal length	438.3	30.1	389.8	440	485.2	0.27	6.86	−0.16	−0.51	0.27	0.43
Shoulder breadth	460.3	23.9	420	460	505.1	0.22	5.20	0.13	−0.39	0.28	0.34
Elbow to elbow length	468.1	32	419.9	470	513.1	0.29	6.83	−0.09	0.19	0.28	0.45
Hip breadth sitting	376.7	29.5	330	375.5	425.3	0.27	7.84	0.12	−0.28	0.23	0.42
Foot breadth	94.2	7.9	81	95	107	0.07	8.40	0.38	−0.04	0.06	0.11
Foot length	249.7	19.3	219.8	250	280	0.18	7.73	0.02	−0.61	0.15	0.27
Weight (kg)	70.63	9.04	57.00	68.00	85.00	0.82	12.79	0.26	−1.07	—	—
BMI (kg/m^2^)	26.09	2.86	21.71	26.02	30.13	0.26	10.95	0.15	−0.03	—	—
RSH	0.51	0.01	0.49	0.51	0.53	0.001	2.88	−0.30	0.09	—	—
BSA (m^2^)	1.77	0.13	1.57	1.76	1.99	0.01	7.58	0.17	−0.06	—	—

All the measurements are in millimeters (mm) except RSH.

**Table 2:** Stature dimension of driver for different countries.

Author/study	Nationalities	Type of vehicle	Stature (mm)
In this study	Bangladesh	Truck and bus	1645
Mazloumi and Mohammadreze [31]	Iran	Shoka	1760
Onawumi and Lucas [32]	Nigeria	Taxicab	1723.8
Kovačević et al. [33]	Croatia	Car	1750
Kyung and Nussbaum [34]	USA	Car	1700
Bose et al. [35]	USA	Motor vehicle	1713.6
Guan et al. [36]	USA	Truck	1745.5
Park et al. [37]	Korea	Car	1662
Zhou et al. [38]	China	NA	1699.2
Mohamad et al. [26]	Malaysia	Car	1687.6

**Table 3:** Comparative study of anthropometry of driver with other nationalities.

Anthropometric measurements	Bangladesh (*n* = 210)	Nigeria (*n* = 1406)	Korea (*n* = 43)	Malaysia (*n* = 708)	China (*n* = 1243)
Stature	1645 ± 70.4	1723.8 ± 63.2	1662 ± 73.2	1687.6 ± 59.43	1699.2
Sitting height erect	843 ± 34.6	834.1 ± 40.2	900 ± 72.7	856.3 ± 61.18	927.9
Sitting shoulder height	589.6 ± 35.8	564.1 ± 45.4	591 ± 28.2	561.6 ± 61.55	NA
Shoulder elbow length	358 ± 26.3	381.5 ± 23.1	329 ± 24	NA	346.6
Thigh clearance height	146.3 ± 18.1	139.3 ± 17.9	NA	NA	144.4
Sitting popliteal height	433.7 ± 25	426.7 ± 26.6	NA	448.1 ± 32.42	421.5
Sitting elbow height	235 ± 21.3	172.5 ± 22	187 ± 26.8	NA	270.8
Elbow fingertip length	461.5 ± 28.4	491.2 ± 42	NA	NA	NA
Buttock popliteal length sitting	438.3 ± 30.1	478.3 ± 39.8	NA	472.6 ± 44.21	NA
Shoulder breadth	460.3 ± 23.9	443.6 ± 28	NA	NA	396.1
Elbow to elbow length	468.1 ± 32	465.1 ± 41.1	NA	NA	401.4
Hip breadth sitting	376.7 ± 29.5	356.6 ± 25.2	341 ± 21.1	367.4 ± 63.06	344.3
Foot breadth	94.2 ± 7.9	99.6 ± 5.5	NA	NA	105
Foot length	249.7 ± 19.3	260.8 ± 13.8	NA	NA	257
Weight	706.3 ± 90.4	NA	627 ± 100.8	NA	NA
BMI	26.09 ± 2.86	NA	NA	NA	NA
RSH	0.51 ± 0.01	NA	NA	NA	NA
BSA	1.77 ± 0.13	NA	NA	NA	NA
